# A Novel Procedure for Knee Flexion Angle Estimation Based on Functionally Defined Coordinate Systems and Independent of the Marker Landmarks

**DOI:** 10.3390/ijerph20010500

**Published:** 2022-12-28

**Authors:** Andrea Ancillao, Arno Verduyn, Maxim Vochten, Erwin Aertbeliën, Joris De Schutter

**Affiliations:** 1 Robotics Research Group, Department of Mechanical Engineering, KU Leuven, 3001 Leuven, Belgium; 2 Core Lab ROB, Flanders Make, KU Leuven, 3001 Leuven, Belgium; 3 Functional Biomechanics and Rehabilitation Engineering Research Unit, Institute of Engineering Design and Product Development, TU Wien, 1060 Vienna, Austria

**Keywords:** biomechanics, gait analysis, invariant, knee angle, knee flexion, motion capture

## Abstract

Knee angles are kinematic quantities that are commonly presented in gait analysis reports. They are typically calculated as the relative angles between the anatomical coordinate systems rigidly attached to the femur and the tibia. To give these angles a biomechanical meaning, the coordinate systems must be defined with respect to some anatomical landmarks. For example, if one axis of the joint coordinate systems is directed along the knee flexion/extension axis, then the relative angle assumes the meaning of flexion/extension angle. Defining accurate anatomical coordinate systems is not an easy task, because it requires skills in marker placement, landmark identification and definition of a biomechanical model. In this paper, we present a novel method to (i) functionally define two coordinate systems attached to femur and tibia and (ii) functionally calculate the knee angle based on the relative differential kinematics between the previously defined coordinate systems. As the main limitation, this method is unable to provide an absolute measurement of the knee flexion/extension angle; however, it is able to accurately capture and display the relative angular motion of the knee. We show that our method produced consistent results even when the measured coordinate systems were randomly modified, removing any anatomical referencing. The proposed method has the advantage of being independent/invariant of the choice of the original coordinate systems of the femur and tibia, removing the need for accurate marker placement. Some major consequences are that (i) the markers may be placed on optimal landmarks, for example, minimizing the soft tissue artifacts or improving the subject’s comfort, and (ii) there is no need for anatomical calibration when technical marker clusters/triads are used.

## 1. Introduction

In the context of clinical motion analysis, it is common practice to study joint kinematics by measuring the joint angles. Such measurements allow one to evaluate the healthiness of a joint, its range of motion and its instantaneous configuration [1,2]. Joint angles are typically presented in the form of a clinical report containing the instantaneous projections of the angles of interest on the three conventional anatomical planes: sagittal, frontal and horizontal [3,4]. For the purpose of clinical assessment, the angles are compared against a control group, typically presented as “normality bands”. Such bands are defined by the mean and standard deviation (SD) of each angular feature as observed in the control group [5,6]. Sometimes, other synthetic descriptors are derived from the joint angles as the root-mean-squared error of the instantaneous differences between the measured features and the respective control features [7,8,9,10]. The angular representation of the motion of a joint is often referred to as “joint kinematics”, and it is considered to be highly informative about the performance of the joint and its possible pathological behaviour [11,12,13]. For example, the pathological condition of crouch gait, which is characterized by the permanent flexion of the knees, can be observed in the gait report as an offset in the knee sagittal angle [7,14,15,16].

The three-dimensional motion of a joint can be recorded by means of motion capture techniques. While the most accurate method would be to use fluoroscopy imaging [17,18], it is not commonly adopted in daily clinical practice due to its bulkiness, difficult setup and possible side effects on the subjects. Instead, the most used non-invasive method is the optoelectronic system, usually installed in a dedicated motion analysis lab [6]. Several alternatives are also possible, such as (i) inertial sensor systems in combination with a biomechanical model [19,20,21], (ii) markerless systems based on computer vision techniques [22] and (iii) other wearable sensors, such as electro-goniometers [23]. Typically, the measurement instrument provides a time series of the poses of the segments composing the joint, which are assumed to be rigid bodies [24]. For example, to describe the motion of the knee, the poses of the femur and of the tibia are recorded and expressed in the form of homogeneous matrices. The kinematic features of the knee are then calculated based on those poses. The three-dimensional knee angles are typically represented in the three anatomical reference planes, i.e., sagittal, frontal and horizontal, and the respective motions are named flexion/extension, abduction/adduction and internal/external rotation [6].

The knee angles are usually obtained via the Euler angle representation of the relative motion between the distal and the proximal segments of the joint [25,26,27]. Such a representation requires the accurate estimation of two anatomically referenced local coordinate systems (CSs), one for the proximal segment and one for the distal segment. Local CSs are typically defined based on some landmarks identified on a biomechanical model, and the coordinate axes are usually aligned to the anatomical rotation axes [25,27,28]. Such CSs are referred to as “anatomical coordinate systems”.

The choice of the marker landmarks, the chosen biomechanical model and, most importantly, the operator’s ability to locate the landmarks have an important effect on the quality and reliability of the measurements [29,30]. For example, it was demonstrated that the knee angles on the frontal and horizontal planes, when measured using an optoelectronic system, are not reliable due to the poor repeatability of marker placement and soft tissue artifact effects [31,32,33]. For this reason, it was recommended to only report the sagittal plane angle in the standard clinical gait report [4,5].

In this paper, we present a novel method for providing an estimation of the knee flexion/extension angle. The novelty of this method is that it does not require a biomechanical model or anatomically defined CSs. Therefore, this method is independent of the chosen marker landmarks, and it does not require a calibration procedure when technical marker clusters are used. The only requirement are the measured coordinates of at least three non-collinear markers attached to each segment composing the knee joint. This method is based on the exploitation of differential kinematics in screw theory and an invariant representation of motion as defined in previous studies [24,34,35].

## 2. Materials and Methods

### 2.1. Calculation of the Knee Angle

The proposed method is based on representing the knee motion around an average screw (helical) axis (ASA), which is functionally determined across a gait cycle [35]. In more detail, the proposed method aims to exploit the knee ASA to define a local coordinate system (CS) relative to a body segment [34,35]. The procedure is repeated twice, once for the femur and once for the tibia, in order to functionally obtain two CSs rigidly attached to the respective body segments. The relative pose of the two CSs can then be used to calculate features of the knee kinematics.

As explained in [24,35], the calculation of the ASA requires knowledge of the differential kinematics of the joint, which can be obtained from the measured poses of femur and tibia, assumed to be available in the form of a time series of homogeneous transformation matrices. In this case, the anatomical calibration of the measured CSs with respect to the respective body segments (assumed to be rigid bodies) is not relevant. In other words, the proposed calculation procedure is independent, or invariant, of the choice of the anatomical CSs. In the following, we refer to these CSs as CS femur and CS tibia, and we use the notation {*fm*} and {*tb*}, respectively.

Naming T0tbi and T0fmi the homogeneous matrices associated with {*tb*} and {*fm*}, with respect to the laboratory CS {0}, at time instant *i*, we can calculate the instantaneous relative pose of {tb} with respect to {*fm*}, represented by the homogeneous transformation matrix as

(1) Tfmtbi=(T0fmi)−1 T0tbi

The differential kinematics of the joint can then be represented by the relative screw twist of {*tb*} with respect to {*fm*}, which can be calculated by differentiating the homogeneous matrix defined in Equation (1) [35,36,37]. The screw twist expresses the relative angular and translational velocity of the knee and can be expressed in either {*fm*} or {*tb*} as follows:

(2) tfmi=(ωfmivfmi) and ttbi=(ωtbivtbi)

The components of the screw twist can be obtained according to the following equations from the homogeneous matrix and the time derivative of the homogeneous matrix:

(3) ([ωfmi]×vfmiO1×30)=T˙fmtbi(Tfmtbi)−1([ωtbi]×vtbiO1×30)=(Tfmtbi)−1T˙fmtbi

where ${\left[\begin{array}{c} \omega_{i}^{} \end{array}\right]}_{\times }$ represents the screw-symmetric matrix defined by vector $\omega_{i}^{}$.

The ASA is represented by a direction vector $n_{\mathrm{ASA}}$ and a point $S_{\mathrm{ASA}}$. These quantities can be calculated with the relative screw twist as defined in Equation (2) using the methods described in [35,38,39,40].

In brief, the direction of the ASA can be obtained as the vector that is “the most parallel” to the set of angular velocities. This can be obtained as the first eigenvector of the covariance matrix of the angular velocity [35] as

(4) $$C_{\omega }=\frac{1}{N}\overset{N}{\underset{i=1}{\sum }}\left(\omega_{i}\omega_{i}^{T}\right)$$

where $\omega_{i}$ corresponds to the rotational component of the screw twist (Equation (2)).

Position $S_{\mathrm{ASA}}$ is calculated as the optimal pseudo-intersection point of the instantaneous screw axes [35].

Based on the ASA calculated from screw twist tfmi, we can define a local CS, attached to the femur, TfmASA_fm, as follows [35]:

origin: = $S_{\mathrm{ASA}}$

${\hat{e}}_{1}$:$\;n_{\mathrm{ASA}}\;$, i.e., the eigenvector belonging to the largest eigenvalue of $C_{\omega }$.

${\hat{e}}_{2}:$ the 2nd eigenvector of $C_{\omega }$.

${\hat{e}}_{3}:{\hat{e}}_{1}\times {\hat{e}}_{2}\;$to ensure a right-handed frame.

The three unit vectors and the origin are assembled in a homogeneous transformation matrix TfmASA_fm.

The procedure is repeated to obtain the local CS TtbASA_tb attached to the tibia, based on screw twist ttbi.

TfmASA_fm and TtbASA_tb are two constant matrices. It is worth to stress that the two CSs are determined in a functional way, only based on the differential kinematics and without the need for anatomical references. The two CSs are located close to the geometric axis of the knee by definition, and *x*-axis (${\hat{e}}_{1}$) lies along the functional rotation axis of the knee, while the other two axes represent the directions in which the principal variations in angular velocity occur [35].

The time-dependent motion of the joint can be represented in terms of the time series of the relative poses between TfmASA_fm and TtbASA_tb, obtained from TfmASA_fm and TtbASA_tb as

(5) TASA_fmASA_tbi=(TfmASA_fm)−1TfmtbiTtbASA_tb

Theses relative poses between the two ASA CSs can be exploited to obtain some clinically meaningful quantities. For example, the orientation part can be represented in terms of Euler angles according to sequence XYZ, and the angle about the *X*-axis represents the functional knee angle. The range of angular displacement along the other two directions gives information about the magnitude of the out-of-plane knee rotations. This procedure is graphically illustrated as a flowchart, provided as supplementary material (File S1).

Since the ASA CSs are defined in a functional way, they are not anatomically referenced. Thus, the calculated angle curves may show an offset that has no physiological meaning. This offset can be removed by setting the initial knee angles to zero, i.e., by subtracting the initial knee angles from the angle curves.

In the next section, we report how we applied this technique to a sample dataset, serving as an example and as demonstration of this method.

### 2.2. Testing on Sample Dataset

The described method was implemented in MATLAB. The method was exploited to calculate the knee angles for a gait analysis trial. For this test, we considered one gait cycle of a gait analysis trial obtained from the CAMS-Knee dataset [41]. The considered trial was subject “K8L”, trial 1. The anatomical poses of {*fm*} and {*tb*}, T0fmi and T0tbi, were recorded using fluoroscopy imaging at 25 Hz [41] and resampled to the gait cycle duration (0–100%) N. Samples = 101. The CS {*fm*} and {*tb*} were defined having the *Z*-axis along the knee rotation axis and the *Y*-axis along the longest dimension of the respective bones. The *X*-axis pointed forward. The gait cycle data were defined as the data between two consecutive heel strikes.

To test the method, we compared each time two sets of Euler angles representing the knee motion. The first set of Euler angles was assumed as the reference anatomical angles. They were calculated according to sequence ZYX from the relative pose of the femur with respect to the tibia as given in Equation (1). The second set of Euler angles corresponded to the invariant knee angles calculated using the method proposed in Section 2.1, starting from the poses of {*fm*} and {*tb*}, T0fmi and T0tbi. The Euler angles associated with the homogeneous matrix shown in Equation (5) were calculated according to sequence XYZ. All the curves were reported after removing the vertical offset from zero. The range of motion of the knee was calculated for each rotation. The ranges of lateral knee motion were reported together as the root-squared sum of the two out-of-plane rotations.

The two sets of knee angles, invariant knee angles and anatomical reference angles were compared, and the root-mean-squared difference between the respective curves was reported.

### 2.3. Test of Invariance

The invariant knee angles were designed to be independent of the position and the orientation of the originally measured CSs. To test the invariance, we modified the measured poses of {*fm*} and {*tb*} by applying an arbitrary rotation and translation, Trnd (different for each CS), as shown in Equation (6). Every anatomical reference for the resulting femur and tibia CSs was lost after this process.

(6) T0fm_rndi=Trnd, 1 T0fmiT0tb_rndi=Trnd,2 T0tbi

The ASA CSs (Equation (5)) were calculated again based on these new poses (Equation (6)). The Euler angles and the ranges of motion were calculated as described in Section 2.2. The results from this case were compared to the previous ones by calculating the root-mean-squared difference between the respective angles.

## 3. Results

**Figure 1:**
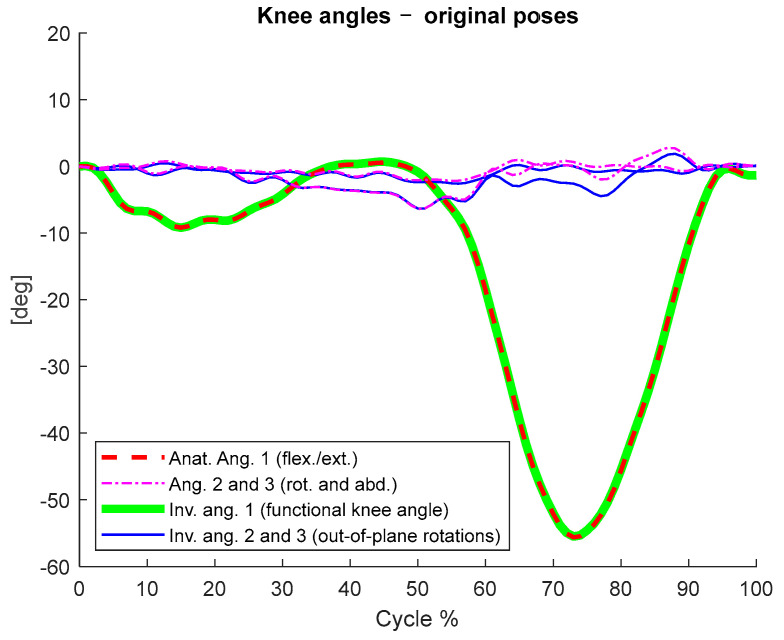
The anatomical angles based on the measured poses and the respective invariant angles calculated according to the proposed procedure.

In Figure 1, we depict the invariant knee angles calculated according to the proposed procedure based on the anatomically referenced CSs and compare them to the Euler angles based on the anatomical jointly referenced CSs.

**Figure 2:**
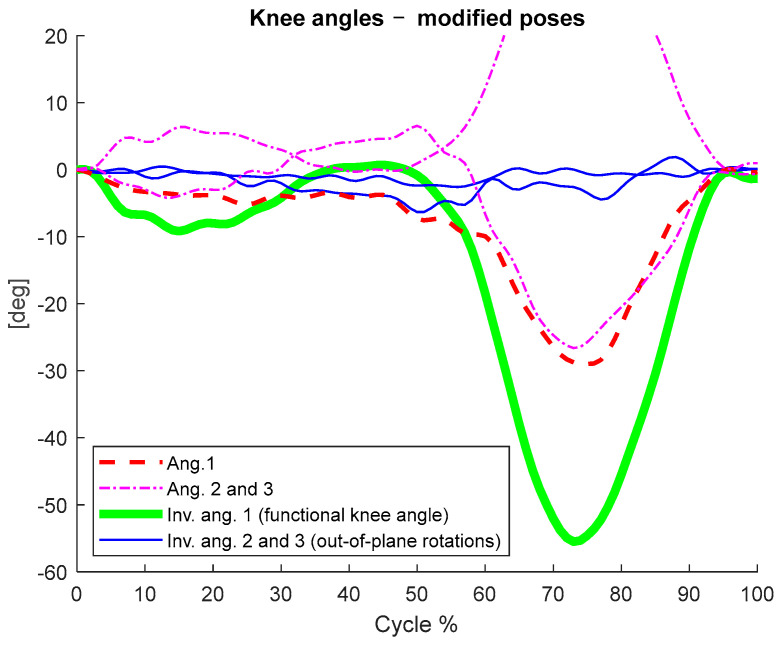
The anatomical angles calculated after applying a random transformation on the measured poses (corresponding to a change in femur and tibia CSs) and the respective invariant angles calculated based on the same modified poses.

In Figure 2, we depict the invariant angles calculated based on the modified CSs, i.e., the CSs translated and rotated by random quantities. In addition, the reference Euler angles between the same modified CSs are reported in the same figure, showing that the curves lost their biomechanical meaning.

For all the curves, we calculated the ranges of motion (RoMs), and these are reported in Table 1. The ranges of motion of Angles 2 and 3 are reported in an aggregated form as the RMS of their sum. We also quantified the RMS differences between (i) the invariant angles and the anatomical angles and (ii) the invariant angles calculated based on different anatomical CSs. All the RMS differences are reported in Table 2.

## 4. Discussion

In this paper, we discuss a novel method for estimating the knee flexion angle. The proposed method has the advantage of being invariant with respect to the choice of the CSs or the marker landmarks chosen to represent the motion of the tibia and the femur. Typically, the knee angles are calculated as the Euler angles between the tibia and femur CSs, which in turn have to be defined and oriented according to anatomical references [6,25,27]. The alignment of the rotation axes with the anatomical directions makes it possible to directly interpret the measured rotations. However, it was shown in the previous literature that even with accurately defined anatomical CSs, the knee rotation and abduction angles are not accurately estimated. The cause was attributed to the relevant error of measurements due to soft tissue artifacts, measurement noise and poor intra-/inter-operator repeatability [31,32]. For this reason, it is recommended to only report the flexion/extension angle in a common gait analysis clinical report.

The methodology proposed in this work allows us to calculate a CS attached to each body segment. Such CS is entirely based on the relative differential kinematics between the segments; thus, it is independent of the chosen anatomical landmarks. The first axis of these functional CSs is the average rotation axis (ASA) of the knee; thus, the angle of rotation about this axis can be interpreted as the knee flexion/extension angle.

With the test conducted on a sample dataset, we showed that the invariant knee angle matched the flexion/extension angle calculated via the anatomical CSs (Figure 1 and Table 2). Furthermore, we showed that the calculation of such an angle and its range of variation were consistent even when the calculation was not based on anatomically referenced measurements (Figure 2 and Table 1). In the invariance test, we randomly translated and rotated the measured CSs. After this procedure, all the anatomical references were lost and the Euler angles calculated based on those CSs lost their clinical meaning (Figure 2 and Table 1, 3rd column). Instead, the functional knee angle calculated according to our invariant method and its range of motion remained coherent with the anatomical conventions, meaning that the clinically relevant information was preserved (Figure 2 and Table 1). The secondary invariant angles (axes 2 and 3) had no anatomical meaning, as the respective axes did not reference any anatomical direction. However, their ranges of motion gave information about the out-of-plane motion of the knee. Such information was consistent with the respective range of motion calculated via the anatomical CSs, is invariant, and can be representative of knee stability [42,43]. However, further studies are needed to assess the correlation among such parameters and some specific pathologies or functional characteristics of the knee.

The main limitation of this method is that being entirely functionally based, it does not measure the absolute knee angle due to the lack of anatomical reference. The consequence is a loss of information about the offset in the curves, as shown in Figure 1 and Figure 2. However, the invariance with respect to anatomical references and location of the markers brings several advantages. For example, (i) accurate marker placement and identification of anatomical landmarks are not required, and the functional CSs and the relative knee angle can be calculated as long as at least three non-collinear marker measurements are available; (ii) other possible choices of marker landmarks become available to improve the subject’s comfort or help to reduce soft tissue artifacts; (iii) accurate measurement of the relative knee angle and its ranges of motion is still possible; (iv) there is the possibility to avoid the anatomical calibration when technical protocols such as CAST [42,44,45] are used.

When the measurement of the absolute knee angles is necessary, e.g., as in [8], the offset can be corrected using an anatomical measurement during the calibration trial, for example, a standing. However, a protocol for such a correction needs to be validated, and its accuracy has to be evaluated in future studies.

## 5. Conclusions

The proposed procedure allows one to estimate the invariant knee flexion/extension angle, i.e., independent of the choice of the anatomical CS representing the motion. The invariant knee angle and its RoM are shown to be coherent with the anatomical ones. The method works with any technical CS or even directly using marker measurements, as long as it is possible to calculate the differential kinematics of the knee joint. Accurate marker placement is not necessary. The main limitation is the lack of anatomical references resulting in missing information about the offset in the knee angle.

## Figures and Tables

**Table 1:** Ranges of motion of the anatomical angles and the invariant angles in the cases of original poses and modified poses.

	Original Poses		Modified Poses	
	Anat. Angle	Invariant Angle	Anat. Angle	Invariant Angle
RoM Angle 1 (deg.)	56.2	56.2	29.1	56.2
RoM Angles 2 + 3 (RMS sum) (deg.)	9.7	8.7	48.3	8.7

**Table 2:** Root-mean-squared differences between (i) the invariant angles and anatomical angles and (ii) the invariant angles calculated based on the original measured poses and the ones calculated based on the randomly modified poses. * Values obtained up to a machine precision of 1 × 10^−13^.

RMS Difference (deg.)	Angle 1	Angle 2	Angle 3
Inv. angles vs. anat. angles	0.1	1.5	2.0
Inv. angles orig. poses vs. Inv. angles mod. poses	0.0 *	0.0 *	0.0 *

## Data Availability

Data is contained within the article.

## Supplementary Materials

The following supporting information can be downloaded at: https://www.mdpi.com/article/10.3390/ijerph20010500/s1, File S1: Flowchart describing the proposed method.
