# Supplementary material for: A Novel Procedure for Knee Flexion Angle Estimation Based on Functionally Defined Coordinate Systems and Independent of the Marker Landmarks
Source: Int J Environ Res Public Health. 2022 Dec 28;20(1):500. doi: 10.3390/ijerph20010500 (PMC9819753; doi:10.3390/ijerph20010500)
Supplement: Supplementary file 1 [file ijerph-20-00500-s001.zip › ijerph-2064141 SI.pdf]

Estimating the invariant knee angle from the measured poses of femur {fm} and tibia {tb}: flowchart of the method.

Annex to:

Ancillao A., Verduyn A., Vochten M., Aertbeliën E., De Schutter J. - A novel procedure for the estimation of knee angles based on functionally defined coordinate systems and independent of the marker landmarks

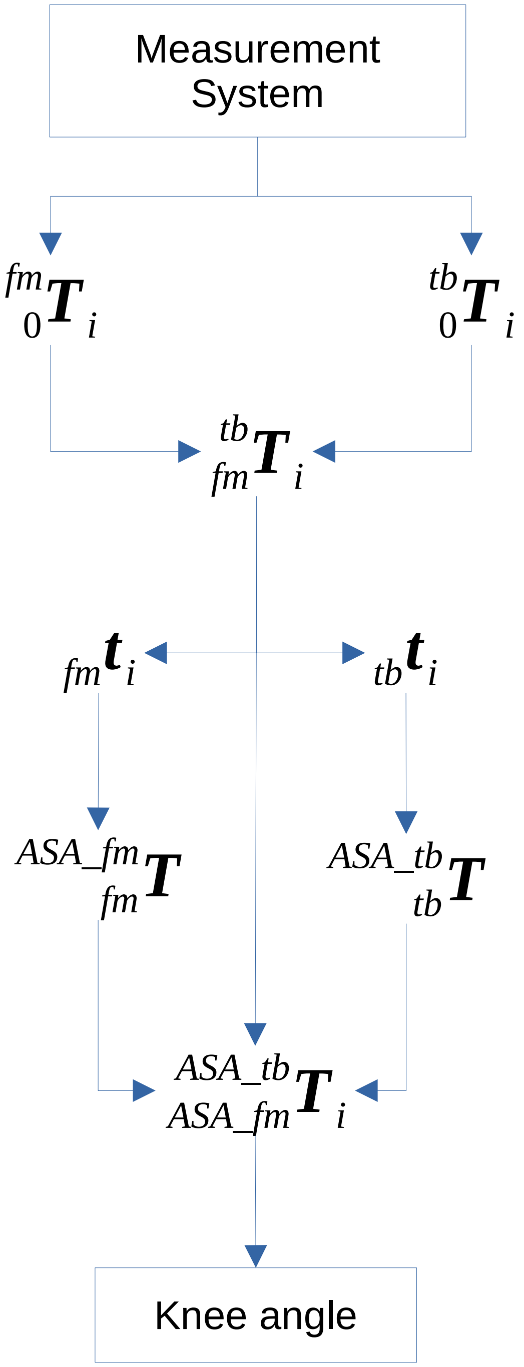

Measured instantaneous poses with respect to the lab. coordinate system {0}.

Relative pose of tibia with respect to femur (eq. 1).

Screw twist of the knee joint seen in {fm} and {tb} (eq. 2 and 3).

ASA Coordinate systems attached to {fm} and {tb}. (Procedure described in [1]).

Relative pose between the two ASA coordinate systems (eq. 5).

Relative orientation of the ASA coordinate systems expressed as Euler angles.

[1] Ancillao et al. 'An Optimal Method for Calculating an Average Screw Axis for a Joint, with Improved Sensitivity to Noise and Providing an Analysis of the Dispersion of the Instantaneous Axes'. PLOS ONE 17, no. 10 (17 October 2022): https://doi.org/10.1371/journal.pone.0275218.
